# Supplementary material for: Simplifying glycan monitoring of complex antigens such as the SARS-CoV-2 spike to accelerate vaccine development
Source: Commun Chem. 2023 Sep 8;6:189. doi: 10.1038/s42004-023-00988-1 (PMC10491790; doi:10.1038/s42004-023-00988-1)
Supplement: Supplementary file 2 — Supplemental Information [file 42004_2023_988_MOESM2_ESM.pdf]

Simplifying glycan monitoring of complex antigens such as the SARS-CoV-2 spike to accelerate vaccine development.

Authors: Janelle Sauvageau[1*], Izel Koyuturk[2,3], Frank St. Michael[1], Denis Brochu[1], Marie-France Goneau[1], Ian Schoenhofen[1], Sylvie Perret[3], Alexandra Star[1], Anna Robotham[1], Arsalan Haqqani[1], John Kelly[1,] Michel Gilbert[1], Yves Durocher[2,3]

Affiliations:

1. Human Health Therapeutics Research Centre, National Research Council of Canada, 100 Sussex Dr., Ottawa, Ontario, K1A 0R6, Canada

2. Department of Biochemistry and Molecular Medicine, Faculty of Medicine, Université de Montréal, Qc, Canada, H3C 3J7.

3. Human Health Therapeutics Research Centre, National Research Council of Canada, 6100 Avenue Royalmount, Montréal, QC, H4P 2R2, Canada

Running title: Monitoring the glycosylation of complex antigens such as the SARS-CoV-2 spike glycoprotein by HPAEC-PAD monosaccharide analysis: helping to accelerate vaccine development

***Corresponding author**: Janelle Sauvageau: Janelle.Sauvageau@nrc-cnrc.gc.ca

Running title: Monosaccharide analysis of SARS-CoV-2 glycoprotein antigens

Table of Contents

Supplementary Sequence 1:Sequence Spike Glycoprotein, putative N-glycosylation sites indicated in yellow 3

Supplementary Table 1. Monosaccharide analysis via HPAEC-PAD of glycovariants of the spike glycoprotein of SARS-CoV-2. 4

Supplementary Table 2. Two way ANOVA with Tukey's test of neutral sugar data for sample 468-472 5

Supplementary Table 3. One way ANOVA with Tukey's test of for the sialic acid analysis of sample 468-472 6

Supplementary Figure 1. Comparison of HILIC-UPLC-Fld chromatograms of PRO-470 (untreated) and PRO-468 after sialidase (MNV) treatment. 8

Supplementary Table 4. List of 27 peptides covering 21 of the 22 spike protein N-linked glycosylation sites that were examined by GlycoPIQ. Enzyme used to generate peptides listed. Glycosylated asparagine indicated with an asterix. 9

Supplementary Table 5. Proportion of biantennary (grey), triantennary (yellow), tetraantennary (blue), hybrid (orange), other glycans (includes glycans such as A1 and A1G, purple) and high-mannose (green). 10

Supplementary Figure 2 Comparison of PRO1-468 and PRO1-471 after sialidase cleavage. 14

Supplementary Table 6: Monosaccharide analysis via HPAEC-PAD of three independent spike batches 15

Supplementary Table 7. Two way ANOVA with Tukey's test of Neutral sugar data for sample PRO1-392, PRO1-394 and PRO1-412 15

Supplementary Table 8: One way ANOVA, of sialic acid data for sample PRO1-392, PRO1-394 and PRO1-412 19

Supplementary Table 9: Depiction of the glycans on 3 independent production batches. Proportion of biantennary (grey), triantennary (yellow), tetraantennary (blue), hybrid (orange), other glycans (includes glycans such as A1 and A1G, purple) and high-mannose (green). 20

Supplementary Table 10: The percentage of galactose as estimated from the LC-MS data. 24

Supplementary Table 11: The percentage of galactose as estimated from the LC-MS data. 25

Supplementary Figure 3. SDS-PAGE Coomassie – Novex gel 4-12%, MES, reducing conditions Final products after buffer exchanged with DPBS pH 7.8. (3 µg per well, Reducing condition: MES buffer, 35 min. at 200 V) 26

Supplementary Table 12. Pool batches characteristics: 27

Supplementary Methods 1. 27

Supplementary Methods 2. 27

Supplementary Methods 3. 28

Supplementary Methods 4. 28

Supplementary Methods 5. 29

Supplementary References :..29

Supplementary Sequence 1:Sequence Spike Glycoprotein, putative N-glycosylation sites indicated in yellow

```
        10         20         30         40         50         60
QCVNLTTRTQ LPPAYTNSFT RGVYYPDKVF RSSVLHSTQD LFLPFFSNVT WFHAIHVSGT

        70         80         90        100        110        120
NGTKRFDNPV LPFNDGVYFA STEKSNIIRG WIFGTTLDSK TQSLLIVNNA TNVVIKVCEF

       130        140        150        160        170        180
QFCNDPFLGV YYHKNNKSWM ESEFRVYSSA NNCTFEYVSQ PFLMDLEGKQ GNFKNLREFV

       190        200        210        220        230        240
FKNIDGYFKI YSKHTPINLV RDLPQGFSAL EPLVDLPIGI NITRFQTLLA LHRSYLTPGD

       250        260        270        280        290        300
SSSGWTAGAA AYYVGYLQPR TFLLKYNENG TITDAVDCAL DPLSETKCTL KSFTVEKGIY

       310        320        330        340        350        360
QTSNFRVQPT ESIVRFPNIT NLCPFGEVFN ATRFASVYAW NRKRISNCVA DYSVLYNSAS

       370        380        390        400        410        420
FSTFKCYGVS PTKLNDLCFT NVYADSFVIR GDEVRQIAPG QTGKIADYNY KLPDDFTGCV

       430        440        450        460        470        480
IAWNSNNLDS KVGGNYNYLY RLFRKSNLKP FERDISTEIY QAGSTPCNGV EGFNCYFPLQ

       490        500        510        520        530        540
SYGFQPTNGV GYQPYRVVVL SFELLHAPAT VCGPKKSTNL VKNKCVNFNF NGLTGTGVLT

       550        560        570        580        590        600
ESNKKFLPFQ QFGRDIADTT DAVRDPQTLE ILDITPCSFG GVSVITPGTN TSNQVAVLYQ

       610        620        630        640        650        660
DVNCTEVPVA IHADQLTPTW RVYSTGSNVF QTRAGCLIGA EHVNNSYECD IPIGAGICAS

       670        680        690        700        710        720
YQTQTNSPGG ASSVASQSII AYTMSLGAEN SVAYSNNSIA IPTNFTISVT TEILPVSMTK

       730        740        750        760        770        780
TSVDCTMYIC GDSTECSNLL LQYGSFCTQL NRALTGIAVE QDKNTQEVFA QVKQIYKTPP

       790        800        810        820        830        840
IKDFGGFNFS QILPDPSKPS KRSFIEDLLF NKVTLADAGF IKQYGDCLGD IAARDLICAQ

       850        860        870        880        890        900
KFNGLTVLPP LLTDEMIAQY TSALLAGTIT SGWTFGAGAA LQIPFAMQMA YRFNGIGVTQ

       910        920        930        940        950        960
NVLYENQKLI ANQFNSAIGK IQDSLSSTAS ALGKLQDVVN QNAQALNTLV KQLSSNFGAI
```

```
       970        980        990       1000       1010       1020
SSVLNDILSR LDPPEAEVQI DRLITGRLQS LQTYVTQQLI RAAEIRASAN LAATKMSECV

      1030       1040       1050       1060       1070       1080
LGQSKRVDFC GKGYHLMSFP QSAPHGVVFL HVTYVPAQEK NFTTAPAICH DGKAHFPREG

      1090       1100       1110       1120       1130       1140
VFVSNGTHWF VTQRNFYEPQ IITTDNTFVS GNCDVVIGIV NNTVYDPLQP ELDSFKEELD

      1150       1160       1170       1180       1190       1200
KYFKNHTSPD VDLGDISGIN ASVVNIQKEI DRLNEVAKNL NESLIDLQEL GKYEQGTGGS

      1210       1220       1230       1240       1250       1260
MEEAINERIQ EVAGSLIFRA ISSIGLECQS VTSRGDLATC PRGFAVTGCT CGSACGSWDV

      1270       1280       1290       1300
RAETTCHCQC AGMDWTGARC CRVQPDYKDD DDKGHHHHHH G
```

Supplementary Table 1. Monosaccharide analysis via HPAEC-PAD of glycovariants of the spike glycoprotein of SARS-CoV-2.

Results reported in mol monosaccharide/mol monomer (Mw = 143,230.6 Da). Results are means of three triplicate injections and three triplicate reactions, the standard deviation is indicated in brackets. [1] TFA hydrolysis (day 1), [2] Enzymatic hydrolysis (day 2). nd: Non-detected Reported with Neu5Gc and Glc content. The values in italics are the % of total glycans in the variant.

	F15 (PRO1-468)	S9 (PRO1-469)	dKO2 (PRO1-470)	WT CHO[BRI/rcTA] (PRO1-471)	WT CHO[BRI/rcTA] (PRO1-472) +kifunensin
Fuc[1]	nd	15.0 (0.8)	nd	15.8 (0.1)	0.8 (0.1)
		6.9%		*6.3%*	*0.3%*
GalN[1]	nd	nd	nd	nd	nd
GlcN[1]	102.5 (0.4)	97.3 (3.5)	88.4 (2.0)	95.6 (0.4)	35.5 (1.0)
	43.0%	*44.7%*	43.9%	*38.0%*	*15.6%*
Gal[1]	43.8 (0.4)	31.6 (1.3)	31.1 (0.5)	43.8 (0.5)	1.7 (0.2)
	18.5%	*14.5%*	*15.4%*	*17.4%*	*0.7%*
Glc[1]	6.1 (0.4)	8.1 (0.6)	17.3 (1.2)	3.9 (0.4)	6.5 (0.2)
	2.5%	*3.7%*	*8.6%*	*1.6%*	*2.8%*
Man[1]	59.2 (1.6)	63.8 (2.3)	62.5 (0.5)	71.1 (0.3)	182.8 (4.8)
	24.9%	*29.3%*	*31.1%*	*28.3%*	*80.0%*
Neu5Ac[2]	26.7 (0.3)	2.0 (0.0)	2.1 (0.0)	21.2 (0.3)	1.3 (0.0)
	11.1%	*0.9%*	*1.0%*	*8.4%*	*0.6%*
Neu5Gc[2]	0.2 (0.0)	0.1 (0.0)	0.0 (0.0)	0.1 (0.0)	0.0 (0.0)
	0.0%	*0.0%*	*0.0%*	*0.0%*	*0.0%*

Supplementary Table 2. Two way ANOVA with Tukey's test of neutral sugar data for sample 468-472

Within each row	compare columns (simple effects within rows)				
Number of families	4				
Number of comparisons per family	10				
Alpha	0.05				
Tukey's multiple comparisons test	Mean Diff.	95.00% CI of diff.	Below threshold?	Summary	Adjusted P Value
Fuc					
PRO-468 vs. PRO-469	-15	-19.59 to -10.41	Yes	****	<0.0001
PRO-468 vs. PRO-470	0	-4.586 to 4.586	No	ns	>0.9999
PRO-468 vs. PRO-471	-15.87	-20.45 to -11.28	Yes	****	<0.0001
PRO-468 vs. PRO-472	-0.7657	-5.352 to 3.820	No	ns	0.989
PRO-469 vs. PRO-470	15	10.41 to 19.59	Yes	****	<0.0001
PRO-469 vs. PRO-471	-0.8667	-5.453 to 3.719	No	ns	0.9826
PRO-469 vs. PRO-472	14.23	9.647 to 18.82	Yes	****	<0.0001
PRO-470 vs. PRO-471	-15.87	-20.45 to -11.28	Yes	****	<0.0001
PRO-470 vs. PRO-472	-0.7657	-5.352 to 3.820	No	ns	0.989
PRO-471 vs. PRO-472	15.1	10.51 to 19.69	Yes	****	<0.0001
GlcN					
PRO-468 vs. PRO-469	5.167	0.5806 to 9.753	Yes	*	0.0204
PRO-468 vs. PRO-470	14.1	9.514 to 18.69	Yes	****	<0.0001
PRO-468 vs. PRO-471	6.867	2.281 to 11.45	Yes	**	0.001
PRO-468 vs. PRO-472	66.97	62.38 to 71.55	Yes	****	<0.0001
PRO-469 vs. PRO-470	8.933	4.347 to 13.52	Yes	****	<0.0001
PRO-469 vs. PRO-471	1.7	-2.886 to 6.286	No	ns	0.8262
PRO-469 vs. PRO-472	61.8	57.21 to 66.39	Yes	****	<0.0001
PRO-470 vs. PRO-471	-7.233	-11.82 to -2.647	Yes	***	0.0005
PRO-470 vs. PRO-472	52.87	48.28 to 57.45	Yes	****	<0.0001
PRO-471 vs. PRO-472	60.1	55.51 to 64.69	Yes	****	<0.0001
Gal					
PRO-468 vs. PRO-469	12.2	7.614 to 16.79	Yes	****	<0.0001
PRO-468 vs. PRO-470	12.73	8.147 to 17.32	Yes	****	<0.0001

PRO-468 vs. PRO-471	0.06667	-4.519 to 4.653	No	ns	>0.9999
PRO-468 vs. PRO-472	42.13	37.55 to 46.72	Yes	****	<0.0001
PRO-469 vs. PRO-470	0.5333	-4.053 to 5.119	No	ns	0.9973
PRO-469 vs. PRO-471	-12.13	-16.72 to -7.547	Yes	****	<0.0001
PRO-469 vs. PRO-472	29.93	25.35 to 34.52	Yes	****	<0.0001
PRO-470 vs. PRO-471	-12.67	-17.25 to -8.081	Yes	****	<0.0001
PRO-470 vs. PRO-472	29.4	24.81 to 33.99	Yes	****	<0.0001
PRO-471 vs. PRO-472	42.07	37.48 to 46.65	Yes	****	<0.0001
Man					
PRO-468 vs. PRO-469	-4.6	-9.186 to -0.01392	Yes	*	0.049
PRO-468 vs. PRO-470	-3.3	-7.886 to 1.286	No	ns	0.2594
PRO-468 vs. PRO-471	-11.97	-16.55 to -7.381	Yes	****	<0.0001
PRO-468 vs. PRO-472	-123.7	-128.3 to -119.1	Yes	****	<0.0001
PRO-469 vs. PRO-470	1.3	-3.286 to 5.886	No	ns	0.9261
PRO-469 vs. PRO-471	-7.367	-11.95 to -2.781	Yes	***	0.0004
PRO-469 vs. PRO-472	-119.1	-123.7 to -114.5	Yes	****	<0.0001
PRO-470 vs. PRO-471	-8.667	-13.25 to -4.081	Yes	****	<0.0001
PRO-470 vs. PRO-472	-120.4	-125.0 to -115.8	Yes	****	<0.0001
PRO-471 vs. PRO-472	-111.7	-116.3 to -107.1	Yes	****	<0.0001

Supplementary Table 3. One way ANOVA with Tukey's test of for the sialic acid analysis of sample 468-472

Number of families	1				
Number of comparisons per family	10				
Alpha	0.05				
Tukey's multiple comparisons test	Mean Diff.	95.00% CI of diff.	Below threshold?	Summary	Adjusted P Value
PRO-468 vs. PRO-469	24.8	23.26 to 26.34	Yes	****	<0.0001
PRO-468 vs. PRO-470	24.6	23.00 to 26.20	Yes	****	<0.0001
PRO-468 vs. PRO-471	5.533	2.819 to 8.248	Yes	*	0.0127
PRO-468 vs. PRO-472	25.43	23.75 to 27.12	Yes	****	<0.0001
PRO-469 vs. PRO-470	-0.2	-0.6442 to 0.2442	No	ns	0.2138

PRO-469 vs. PRO-471	-19.27	-21.12 to -17.42	Yes	****	<0.0001
PRO-469 vs. PRO-472	0.6333	0.3769 to 0.8898	Yes	**	0.009
PRO-470 vs. PRO-471	-19.07	-20.49 to -17.64	Yes	****	<0.0001
PRO-470 vs. PRO-472	0.8333	0.5769 to 1.090	Yes	**	0.0049
PRO-471 vs. PRO-472	19.9	18.30 to 21.50	Yes	****	<0.0001

Supplementary Figure 1. Comparison of HILIC-UPLC-Fld chromatograms of PRO-470 (untreated) and PRO-468 after sialidase (MNV) treatment.

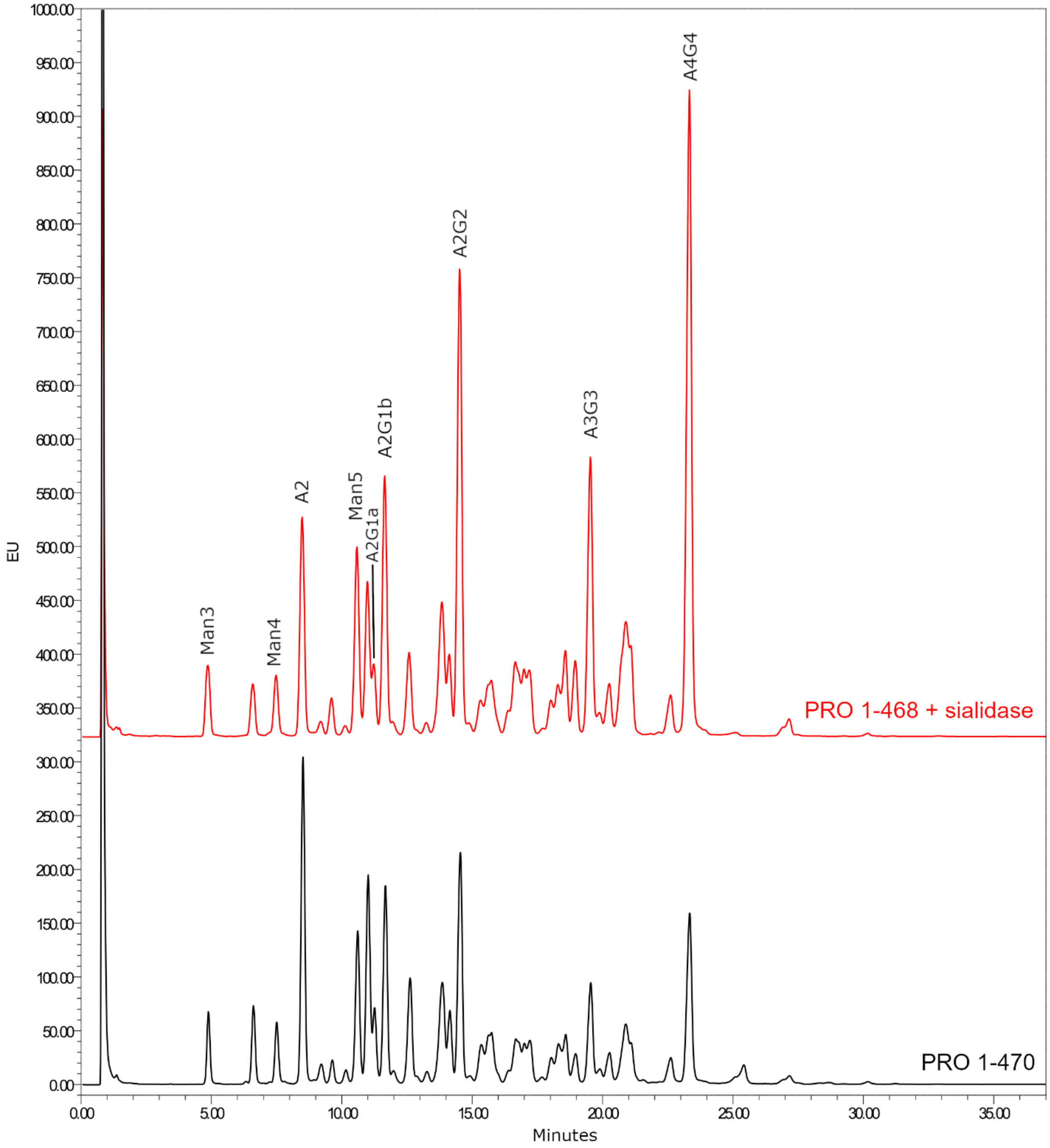

Supplementary Table 4. List of 27 peptides covering 21 of the 22 spike protein N-linked glycosylation sites that were examined by GlycoPIQ. Enzyme used to generate peptides listed. Glycosylated asparagine indicated with an asterix.

Site	Peptide and N-linked site	Proteolytic Enzyme
N17	QCVN*LTTR	Trypsin
N61	FSN*VT	a-Lytic
N61	QDLFLPFFSN*VT	a-Lytic
N61	FSN*VTWF	Chymotrypsin
N74	GTN*GTKRFDNPVLPFNDGVYFA	a-Lytic
N122	TQSLLIVNN*ATNVVIK	Trypsin
N149	FQFCNDPFLGVYYHKNN*KSWME	GluC
N165	FRVYSSANN*CTFE	GluC
N165	VYSSANN*CTFEYVSQPFLMDLEGK	Trypsin
N234	DLPQGFSALEPLVDLPIGIN*ITR	Trypsin
N282	YNEN*GTITDAVDCALDPLSETK	Trypsin
N331	SIVRFPN*ITNLCPFGE	GluC
N343	NLCPFGEVFN*AT	a-Lytic
N603	VITPGTN*TS	a-Lytic
N616	VLYQDVN*CT	a-Lytic
N616	LYQDVN*CT	a-Lytic
N657	AEHVN*N*SYECDIPI	a-Lytic
N657	N*N*SYECDIPI	a-Lytic
N709	YSN*N*SIAIPT	a-Lytic
N717	N*FTIS	a-Lytic
N801	DFGGFN*FSQILPDPSKPSK	Trypsin
N1074	N*FTTAPAICHDGK	Trypsin
N1098	EGVFVSN*GTHWFVTQR	Trypsin
N1158	LDKYFKN*HTSPDVD	GluC
N1173	GIN*ASVV	a-Lytic
N1173	LGDISGIN*ASVVN*IQKE	GluC
N1194	N*LN*ESLIDLQELGK	Trypsin

Supplementary Table 5. Proportion of biantennary (grey), triantennary (yellow), tetraantennary (blue), hybrid (orange), other glycans (includes glycans such as A1 and A1G, purple) and high-mannose (green).

	PRO-468	PRO-469	PRO-470	PRO-471	PRO-472
N-17 Tr	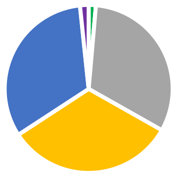	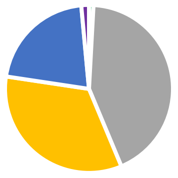	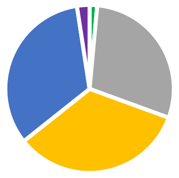	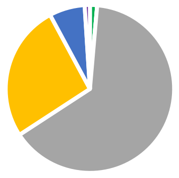	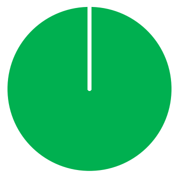
N-61 AL1	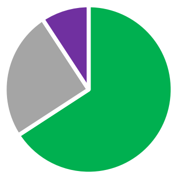	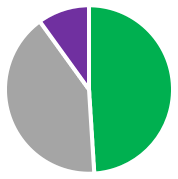	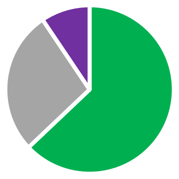	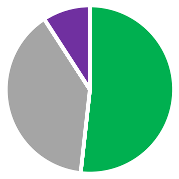	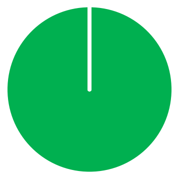
N-61 AL2	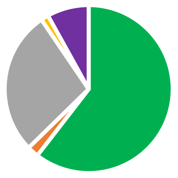	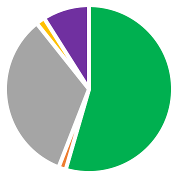	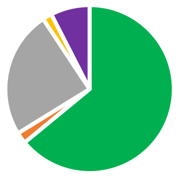	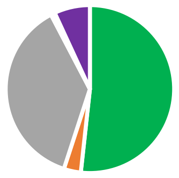	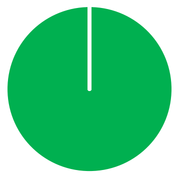
N-61 CH	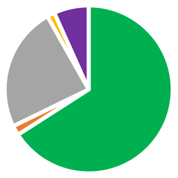	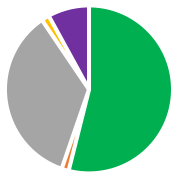	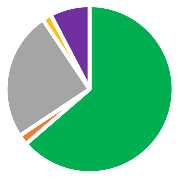	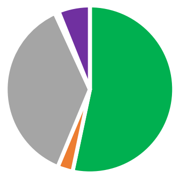	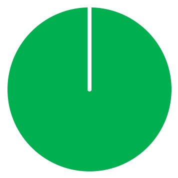
N-74 AL	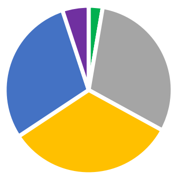	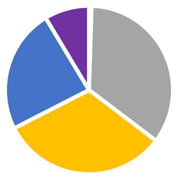	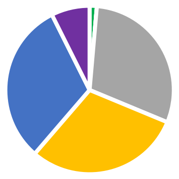	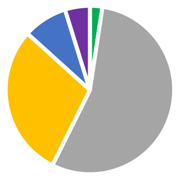	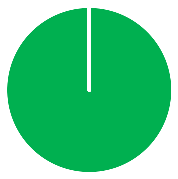
N-122 TR	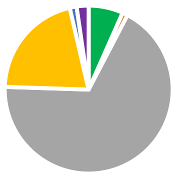	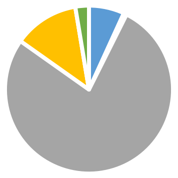	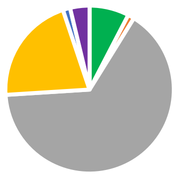	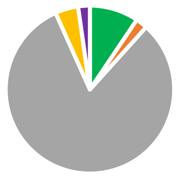	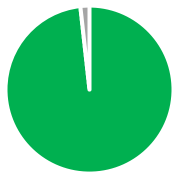

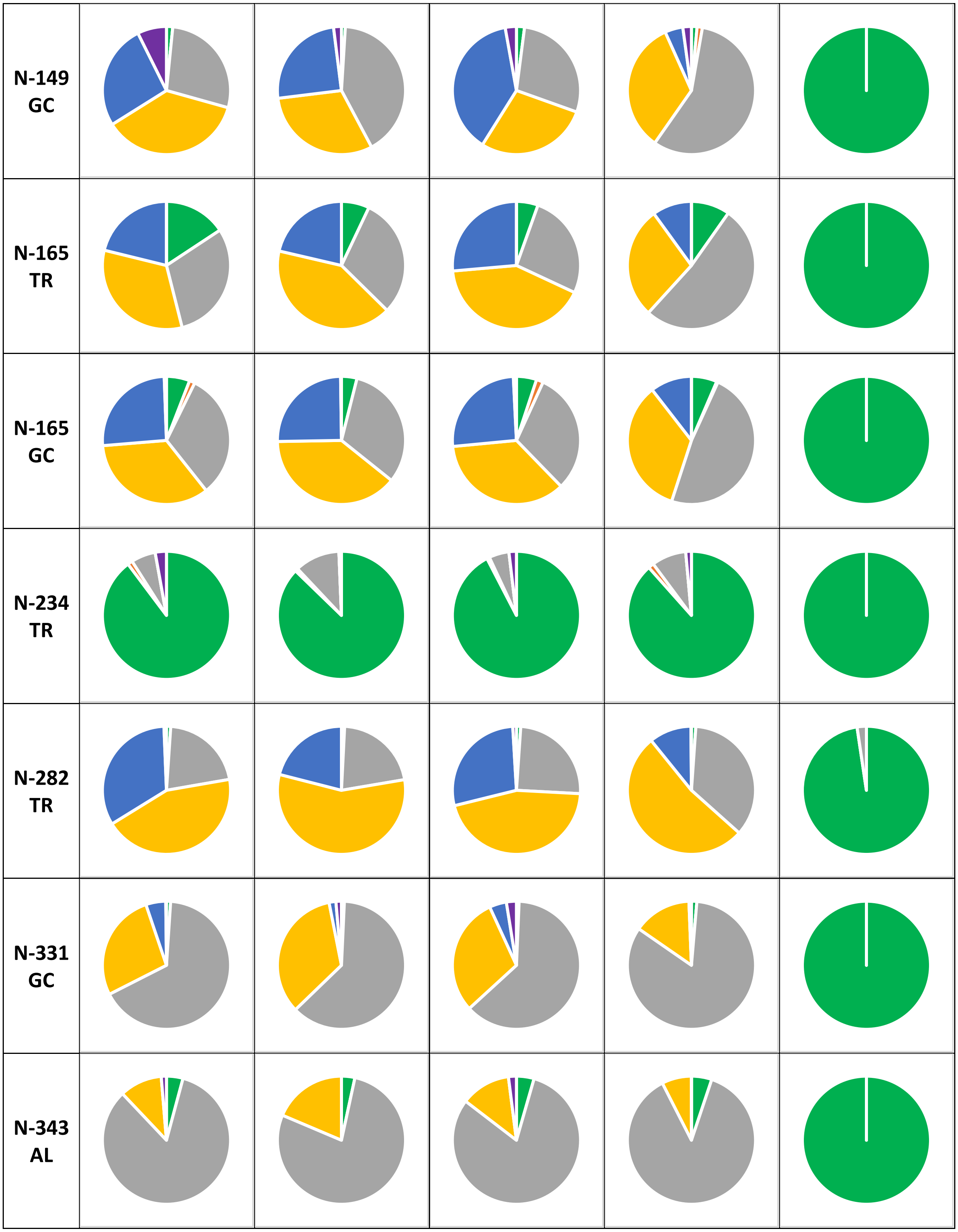
N-149
GC
N-165
TR
N-165
GC
N-234
TR
N-282
TR
N-331
GC
N-343
AL

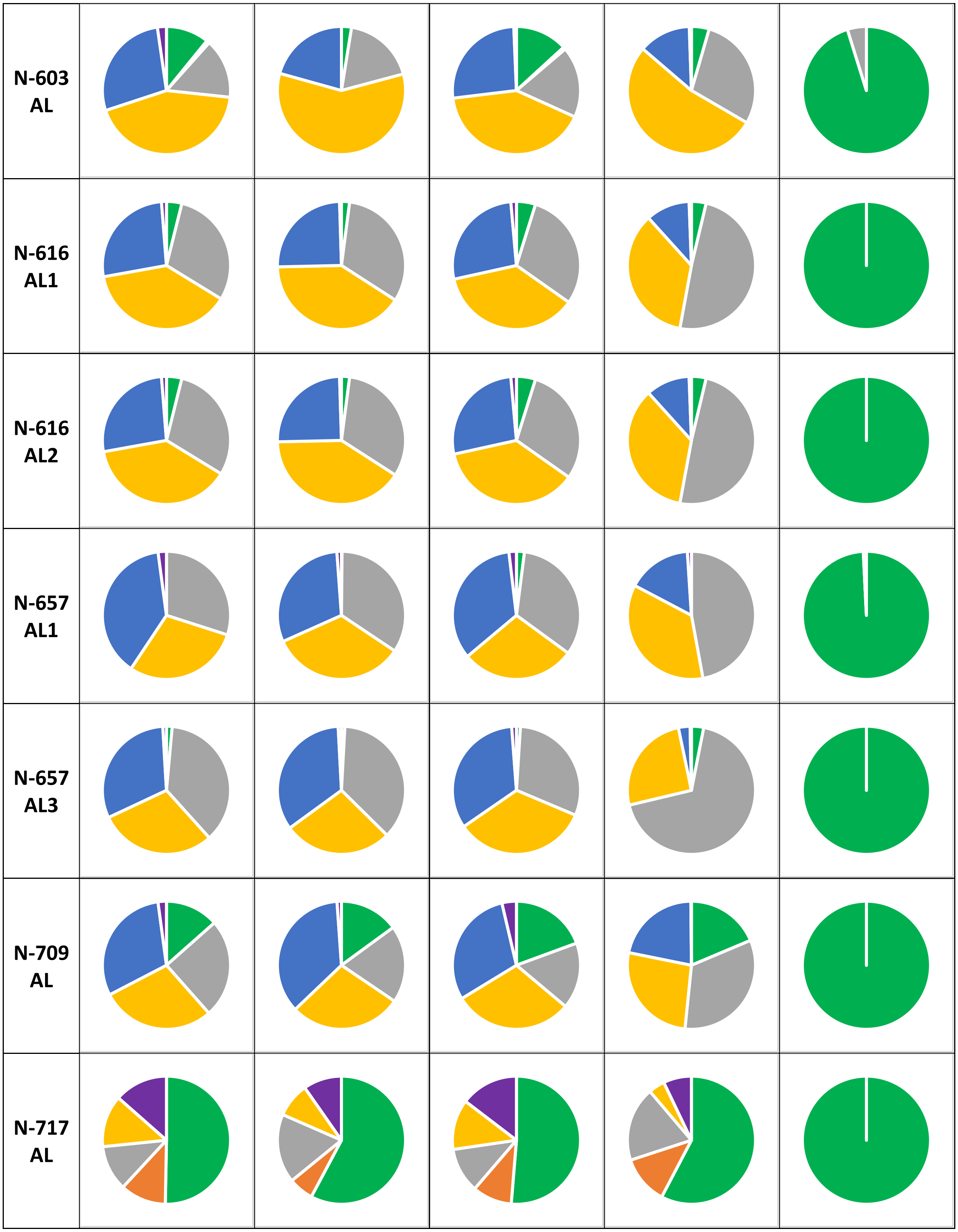
N-603
AL
N-616
AL1
N-616
AL2
N-657
AL1
N-657
AL3
N-709
AL
N-717
AL

**N801 TR**	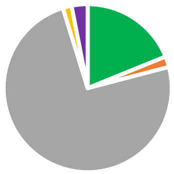	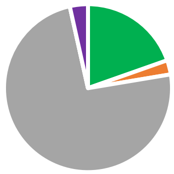	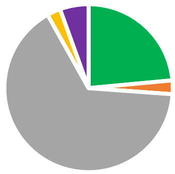	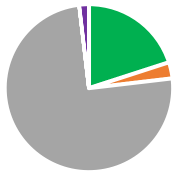	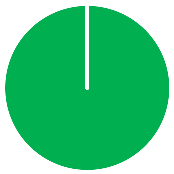
**N-1074 TR**	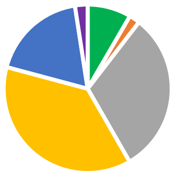	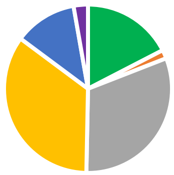	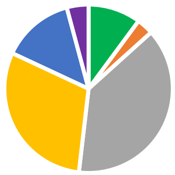	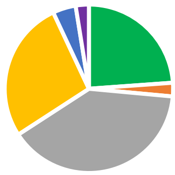	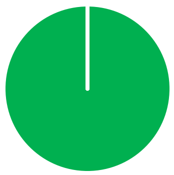
**N-1098 TR**	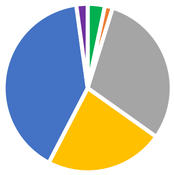	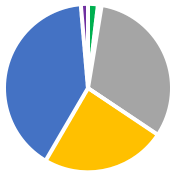	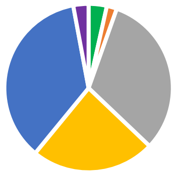	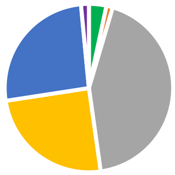	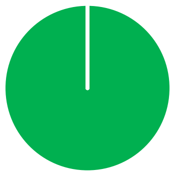
**N-1158 GC**	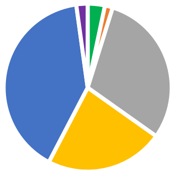	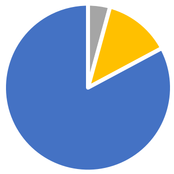	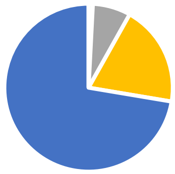	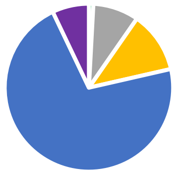	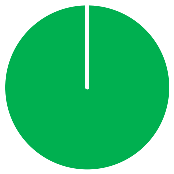
**N-1173 GC**	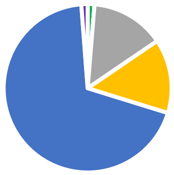	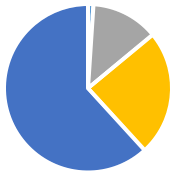	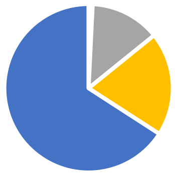	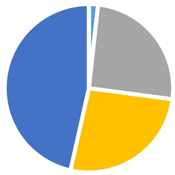	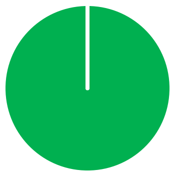
**N-1173 AL**	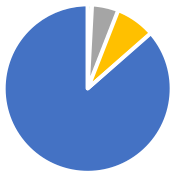	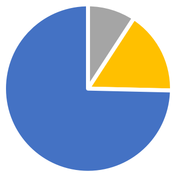	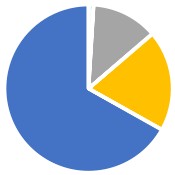	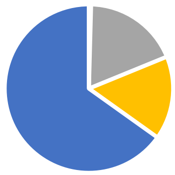	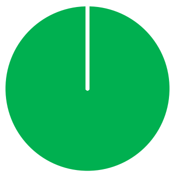
**N-1174 TR**	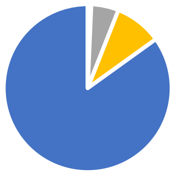	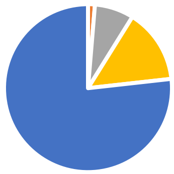	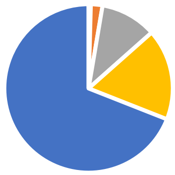	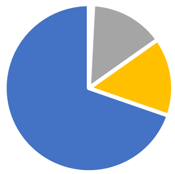	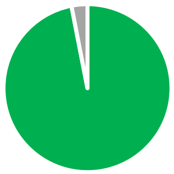

Supplementary Figure 2 Comparison of PRO1-468 and PRO1-471 after sialidase cleavage.

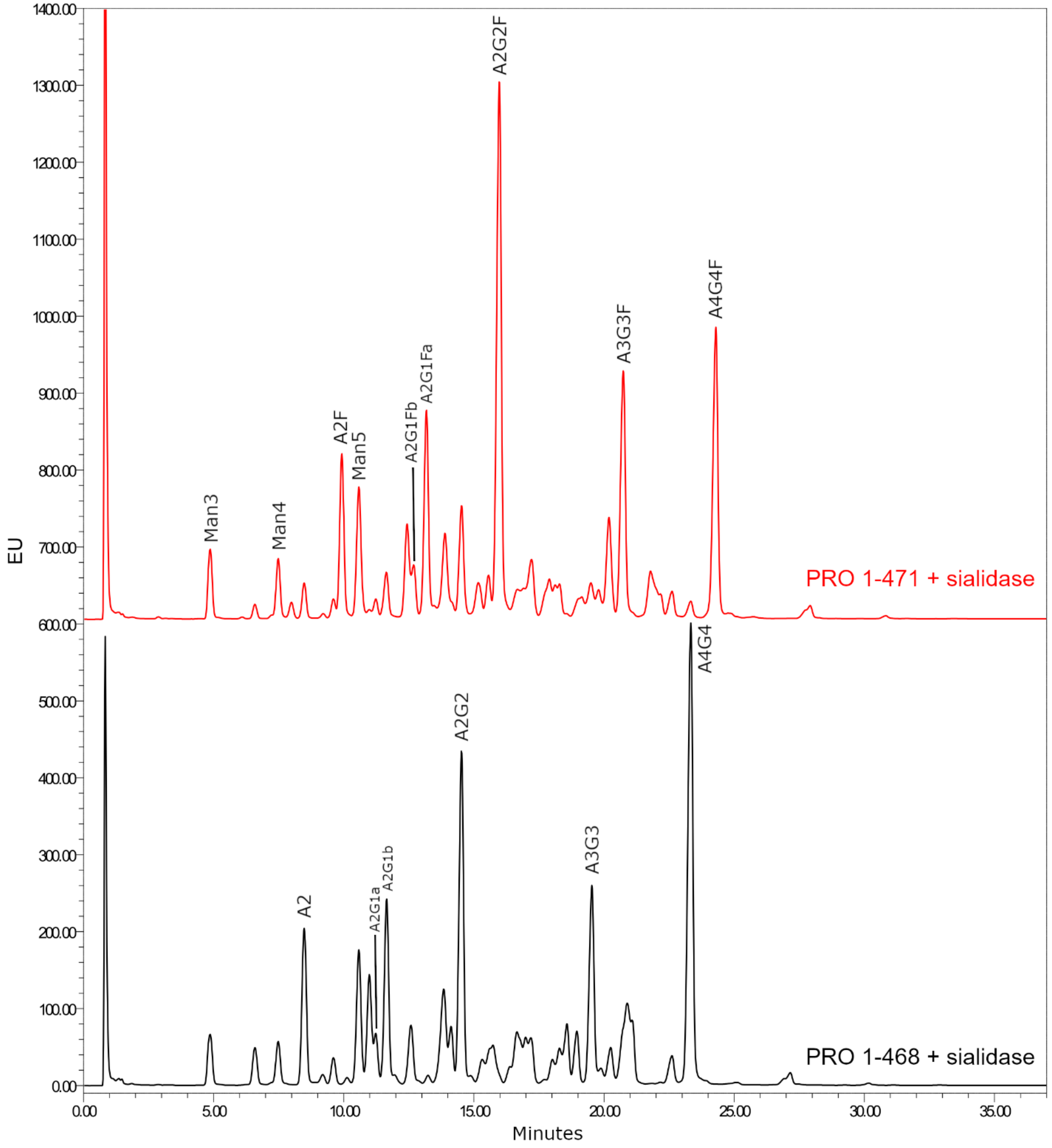

Supplementary Table 6: Monosaccharide analysis via HPAEC-PAD of three independent spike batches

Results reported in mol monosaccharide/mol monomer (Mw = 143,230.6 Da). Results are means of three triplicate injections and three triplicate reactions, the standard deviation is indicated in brackets. [1] TFA hydrolysis (day 3), [2] Enzymatic hydrolysis (day 4), nd: Non-detected. Reported with Neu5Gc and Glc content.

	PRO1-392	PRO1-394	PRO1-412
Fuc[1]	12.3 (0.7)	10.2 (0.5)	9.4 (0.5)
GalN[1]	0.0 (0.0)	0.0 (0.0)	nd
GlcN[1]	72.8 (4.2)	61.1 (2.8)	58.1 (3.2)
Gal[1]	38.0 (2.4)	26.8 (1.2)	25.8 (1.4)
Glc[1]	0.0 (0.0)	0.0 (0.0)	0.0 (0.0)
Man[1]	54.0 (3.2)	53.0 (2.6)	50.5 (3.5)
Neu5Ac[2]	16.5 (0.1)	10.8 (0.2)	8.7 (0.5)
Neu5Gc[2]	0.1 (0.0)	0.1 (0.0)	0.1 (0.0)

Supplementary Table 7. Two way ANOVA with Tukey's test of Neutral sugar data for sample PRO1-392, PRO1-394 and PRO1-412

Number of families	1	**Compare cell means regardless of rows and columns**			
Number of comparisons per family	66				
Alpha	0.05				
Tukey's multiple comparisons test	Mean Diff.	95.00% CI of diff.	Below threshold?	Summary	Adjusted P Value
Fuc:PRO1-392 vs. Fuc:PRO1-394	2.133	-6.981 to 11.25	No	ns	0.9991
Fuc:PRO1-392 vs. Fuc:PRO1-412	2.867	-6.248 to 11.98	No	ns	0.9895
Fuc:PRO1-392 vs. GlcN:PRO1-392	-60.53	-69.65 to -51.42	Yes	****	<0.0001
Fuc:PRO1-392 vs. GlcN:PRO1-394	-48.83	-57.95 to -39.72	Yes	****	<0.0001
Fuc:PRO1-392 vs. GlcN:PRO1-412	-45.83	-54.95 to -36.72	Yes	****	<0.0001
Fuc:PRO1-392 vs. Gal:PRO1-392	-25.67	-34.78 to -16.55	Yes	****	<0.0001

Fuc:PRO1-392 vs. Gal:PRO1-394	-14.5	-23.61 to -5.386	Yes	***	0.0003
Fuc:PRO1-392 vs. Gal:PRO1-412	-13.43	-22.55 to -4.319	Yes	***	0.0009
Fuc:PRO1-392 vs. Man:PRO1-392	-41.7	-50.81 to -32.59	Yes	****	<0.0001
Fuc:PRO1-392 vs. Man:PRO1-394	-40.73	-49.85 to -31.62	Yes	****	<0.0001
Fuc:PRO1-392 vs. Man:PRO1-412	-38.23	-47.35 to -29.12	Yes	****	<0.0001
Fuc:PRO1-394 vs. Fuc:PRO1-412	0.7333	-8.381 to 9.848	No	ns	>0.9999
Fuc:PRO1-394 vs. GlcN:PRO1-392	-62.67	-71.78 to -53.55	Yes	****	<0.0001
Fuc:PRO1-394 vs. GlcN:PRO1-394	-50.97	-60.08 to -41.85	Yes	****	<0.0001
Fuc:PRO1-394 vs. GlcN:PRO1-412	-47.97	-57.08 to -38.85	Yes	****	<0.0001
Fuc:PRO1-394 vs. Gal:PRO1-392	-27.8	-36.91 to -18.69	Yes	****	<0.0001
Fuc:PRO1-394 vs. Gal:PRO1-394	-16.63	-25.75 to -7.519	Yes	****	<0.0001
Fuc:PRO1-394 vs. Gal:PRO1-412	-15.57	-24.68 to -6.452	Yes	***	0.0001
Fuc:PRO1-394 vs. Man:PRO1-392	-43.83	-52.95 to -34.72	Yes	****	<0.0001
Fuc:PRO1-394 vs. Man:PRO1-394	-42.87	-51.98 to -33.75	Yes	****	<0.0001
Fuc:PRO1-394 vs. Man:PRO1-412	-40.37	-49.48 to -31.25	Yes	****	<0.0001
Fuc:PRO1-412 vs. GlcN:PRO1-392	-63.4	-72.51 to -54.29	Yes	****	<0.0001
Fuc:PRO1-412 vs. GlcN:PRO1-394	-51.7	-60.81 to -42.59	Yes	****	<0.0001
Fuc:PRO1-412 vs. GlcN:PRO1-412	-48.7	-57.81 to -39.59	Yes	****	<0.0001
Fuc:PRO1-412 vs. Gal:PRO1-392	-28.53	-37.65 to -19.42	Yes	****	<0.0001
Fuc:PRO1-412 vs. Gal:PRO1-394	-17.37	-26.48 to -8.252	Yes	****	<0.0001
Fuc:PRO1-412 vs. Gal:PRO1-412	-16.3	-25.41 to -7.186	Yes	****	<0.0001

Fuc:PRO1-412 vs. Man:PRO1-392	-44.57	-53.68 to -35.45	Yes	****	<0.0001
Fuc:PRO1-412 vs. Man:PRO1-394	-43.6	-52.71 to -34.49	Yes	****	<0.0001
Fuc:PRO1-412 vs. Man:PRO1-412	-41.1	-50.21 to -31.99	Yes	****	<0.0001
GlcN:PRO1-392 vs. GlcN:PRO1-394	11.7	2.586 to 20.81	Yes	**	0.0048
GlcN:PRO1-392 vs. GlcN:PRO1-412	14.7	5.586 to 23.81	Yes	***	0.0003
GlcN:PRO1-392 vs. Gal:PRO1-392	34.87	25.75 to 43.98	Yes	****	<0.0001
GlcN:PRO1-392 vs. Gal:PRO1-394	46.03	36.92 to 55.15	Yes	****	<0.0001
GlcN:PRO1-392 vs. Gal:PRO1-412	47.1	37.99 to 56.21	Yes	****	<0.0001
GlcN:PRO1-392 vs. Man:PRO1-392	18.83	9.719 to 27.95	Yes	****	<0.0001
GlcN:PRO1-392 vs. Man:PRO1-394	19.8	10.69 to 28.91	Yes	****	<0.0001
GlcN:PRO1-392 vs. Man:PRO1-412	22.3	13.19 to 31.41	Yes	****	<0.0001
GlcN:PRO1-394 vs. GlcN:PRO1-412	3	-6.114 to 12.11	No	ns	0.9851
GlcN:PRO1-394 vs. Gal:PRO1-392	23.17	14.05 to 32.28	Yes	****	<0.0001
GlcN:PRO1-394 vs. Gal:PRO1-394	34.33	25.22 to 43.45	Yes	****	<0.0001
GlcN:PRO1-394 vs. Gal:PRO1-412	35.4	26.29 to 44.51	Yes	****	<0.0001
GlcN:PRO1-394 vs. Man:PRO1-392	7.133	-1.981 to 16.25	No	ns	0.2318
GlcN:PRO1-394 vs. Man:PRO1-394	8.1	-1.014 to 17.21	No	ns	0.1145
GlcN:PRO1-394 vs. Man:PRO1-412	10.6	1.486 to 19.71	Yes	*	0.0134
GlcN:PRO1-412 vs. Gal:PRO1-392	20.17	11.05 to 29.28	Yes	****	<0.0001
GlcN:PRO1-412 vs. Gal:PRO1-394	31.33	22.22 to 40.45	Yes	****	<0.0001
GlcN:PRO1-412 vs. Gal:PRO1-412	32.4	23.29 to 41.51	Yes	****	<0.0001

GlcN:PRO1-412 vs. Man:PRO1-392	4.133	-4.981 to 13.25	No	ns	0.8792
GlcN:PRO1-412 vs. Man:PRO1-394	5.1	-4.014 to 14.21	No	ns	0.6786
GlcN:PRO1-412 vs. Man:PRO1-412	7.6	-1.514 to 16.71	No	ns	0.167
Gal:PRO1-392 vs. Gal:PRO1-394	11.17	2.052 to 20.28	Yes	**	0.0079
Gal:PRO1-392 vs. Gal:PRO1-412	12.23	3.119 to 21.35	Yes	**	0.0029
Gal:PRO1-392 vs. Man:PRO1-392	-16.03	-25.15 to -6.919	Yes	****	<0.0001
Gal:PRO1-392 vs. Man:PRO1-394	-15.07	-24.18 to -5.952	Yes	***	0.0002
Gal:PRO1-392 vs. Man:PRO1-412	-12.57	-21.68 to -3.452	Yes	**	0.0021
Gal:PRO1-394 vs. Gal:PRO1-412	1.067	-8.048 to 10.18	No	ns	>0.9999
Gal:PRO1-394 vs. Man:PRO1-392	-27.2	-36.31 to -18.09	Yes	****	<0.0001
Gal:PRO1-394 vs. Man:PRO1-394	-26.23	-35.35 to -17.12	Yes	****	<0.0001
Gal:PRO1-394 vs. Man:PRO1-412	-23.73	-32.85 to -14.62	Yes	****	<0.0001
Gal:PRO1-412 vs. Man:PRO1-392	-28.27	-37.38 to -19.15	Yes	****	<0.0001
Gal:PRO1-412 vs. Man:PRO1-394	-27.3	-36.41 to -18.19	Yes	****	<0.0001
Gal:PRO1-412 vs. Man:PRO1-412	-24.8	-33.91 to -15.69	Yes	****	<0.0001
Man:PRO1-392 vs. Man:PRO1-394	0.9667	-8.148 to 10.08	No	ns	>0.9999
Man:PRO1-392 vs. Man:PRO1-412	3.467	-5.648 to 12.58	No	ns	0.9585
Man:PRO1-394 vs. Man:PRO1-412	2.5	-6.614 to 11.61	No	ns	0.9965

Supplementary Table 8: One way ANOVA, of sialic acid data for sample PRO1-392, PRO1-394 and PRO1-412

Number of families	1					
Number of comparisons per family	3					
Alpha	0.05					
Tukey's multiple comparisons test	Mean Diff.	95.00% CI of diff.	Below threshold?	Summary	Adjusted P Value	
PRO1-392 vs. PRO1-394	5.667	4.791 to 6.543	Yes	****	<0.0001	A-B
PRO1-392 vs. PRO1-412	7.733	6.857 to 8.609	Yes	****	<0.0001	A-C
PRO1-394 vs. PRO1-412	2.067	1.191 to 2.943	Yes	***	0.0009	B-C

Supplementary Table 9: Depiction of the glycans on 3 independent production batches. Proportion of biantennary (grey), triantennary (yellow), tetraantennary (blue), hybrid (orange), other glycans (includes glycans such as A1 and A1G, purple) and high-mannose (green).

	PRO1-394	PRO-412	PRO-392
N-17 Tr	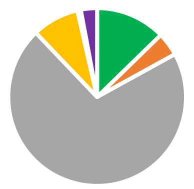	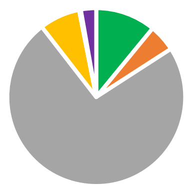	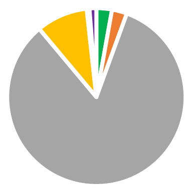
N-61 AL1	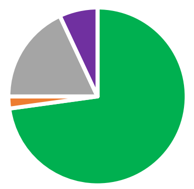	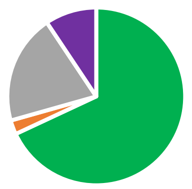	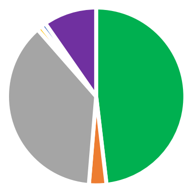
N-61 AL2	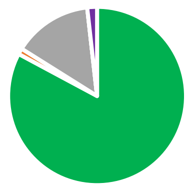	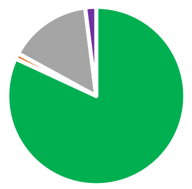	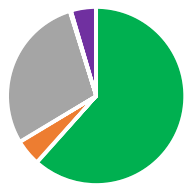
N-61 CH	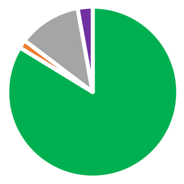	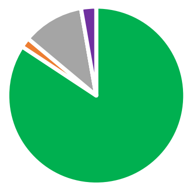	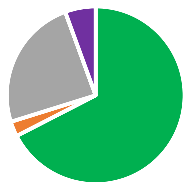
N-74 AL	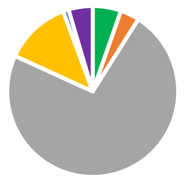	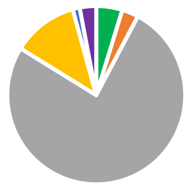	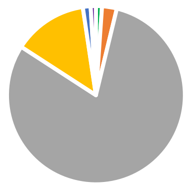
N-122 TR	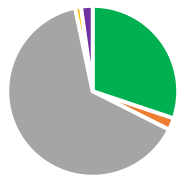	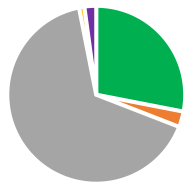	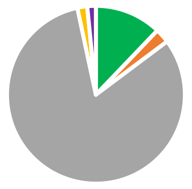

**N-149 GC**	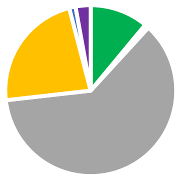	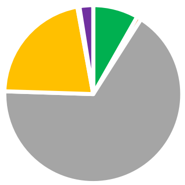	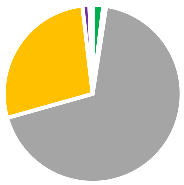
**N-165 TR**	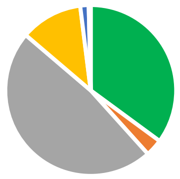	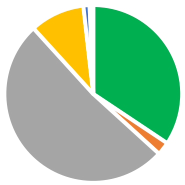	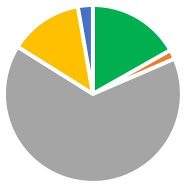
**N-165 GC**	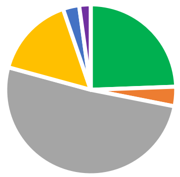	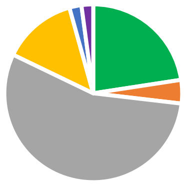	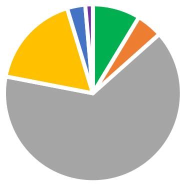
**N-234 TR**	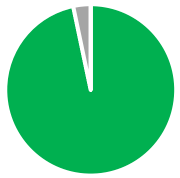	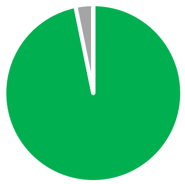	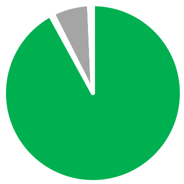
**N-282 TR**	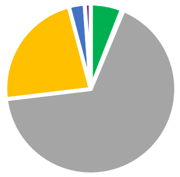	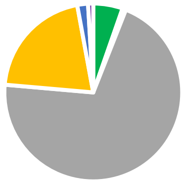	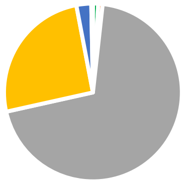
**N-331 GC**	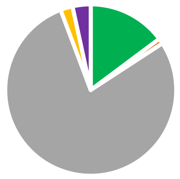	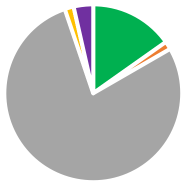	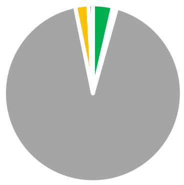
**N-343 AL**	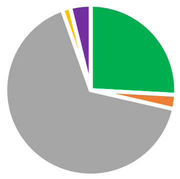	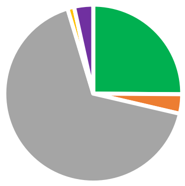	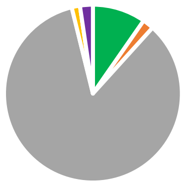

**N-603 AL**	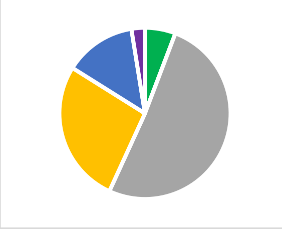	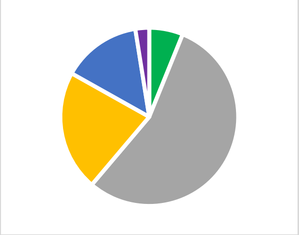	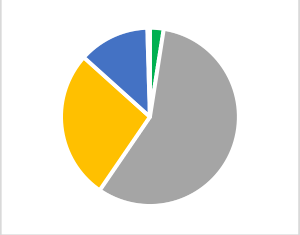
**N-616 AL1**	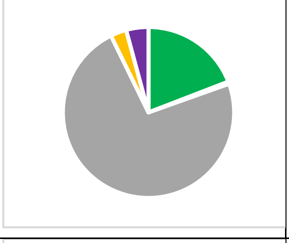	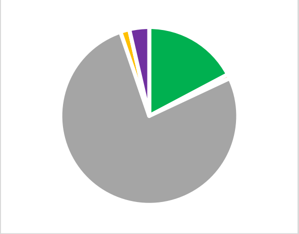	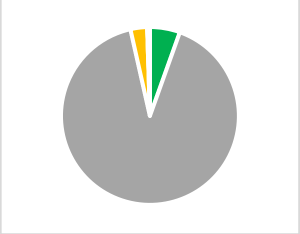
**N-616 AL2**	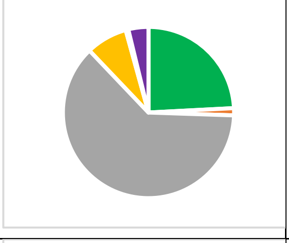	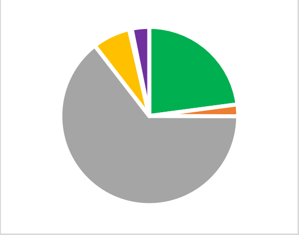	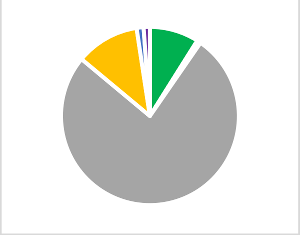
**N-657 AL1**	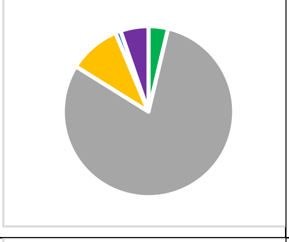	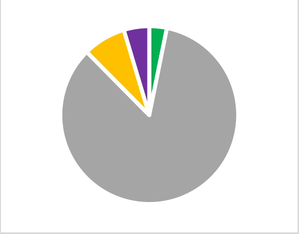	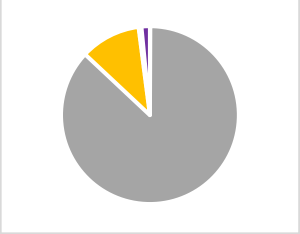
**N-657 AL3**	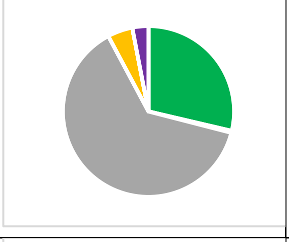	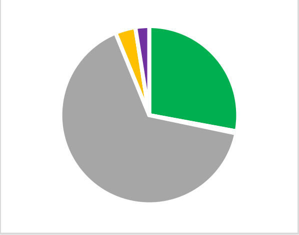	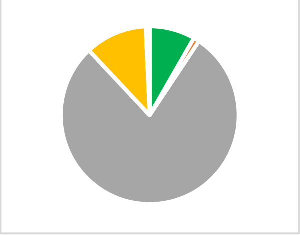
**N-717 AL**	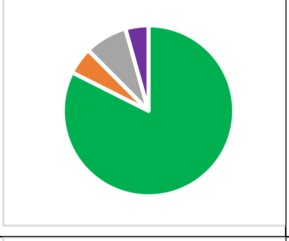	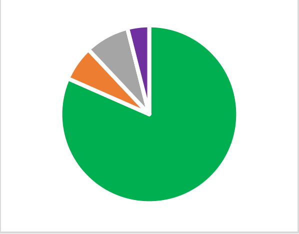	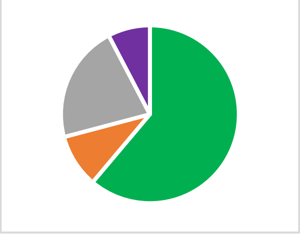
**N801 TR**	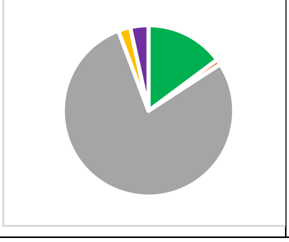	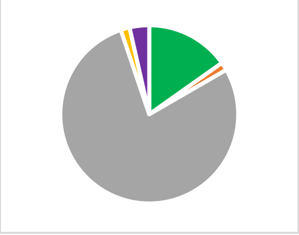	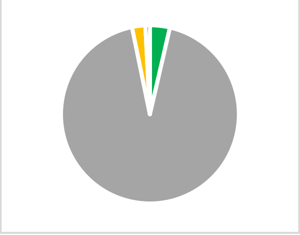

**N-1074 TR**	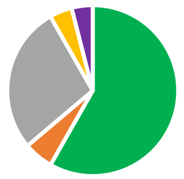	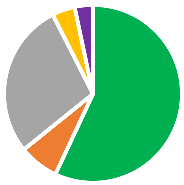	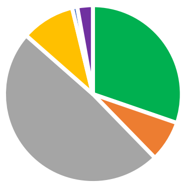
**N-1098 TR**	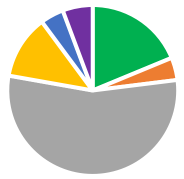	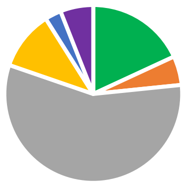	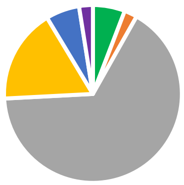
**N-1158 GC**	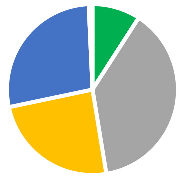	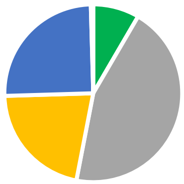	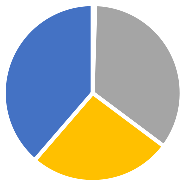
**N-1173 AL**	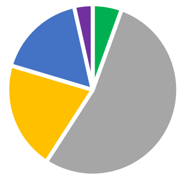	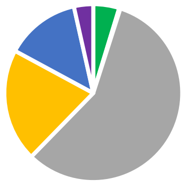	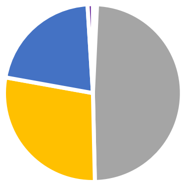
**N-1174 TR**	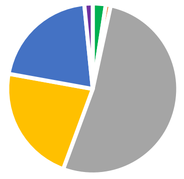	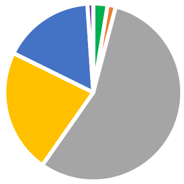	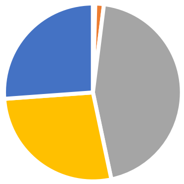

Supplementary Table 10: The percentage of galactose as estimated from the LC-MS data.

The apparent relative abundance of each glycan per site was multiplied by the number of HexNac, Man, Gal, Fuc and Neu5Ac residues per glycan. After averaging all sites, the relative % of Gal was calculated from the sum of all the monosaccharides. Please note that this is not a quantitative measure of galactose content.

	***N*-glycans containing galactose**		
	**PRO1-392**	**PRO1-412**	**PRO1-394**
N17_Tr	17.46%	15.75%	15.17%
N61_aL1	11.46%	6.68%	5.86%
N61_aL2	8.35%	4.08%	3.92%
N61_Ch	7.22%	3.48%	3.52%
N61_Average	9.02%	4.82%	4.42%
N74_aL	18.15%	17.55%	17.13%
N122_Tr	16.95%	13.89%	13.12%
N149_GC	19.46%	17.85%	17.22%
N165_Tr	16.60%	14.03%	14.16%
N165_GC	18.15%	16.05%	16.01%
N165_Average	17.38%	15.06%	15.10%
N234_Tr	0.89%	0.25%	0.22%
N282_Tr	19.09%	17.25%	16.42%
N331_GC	17.05%	14.92%	14.59%
N343_aL	17.08%	14.46%	14.17%
N603_aL	18.45%	16.06%	15.41%
N616_aL1	17.34%	14.32%	13.41%
N616_aL2	17.69%	14.15%	13.19%
N616_Average	17.51%	14.24%	13.30%
N657_aL1	17.83%	16.55%	16.06%
N657_aL3	17.27%	13.48%	12.50%
N657_Average	17.56%	15.11%	14.40%
N717_aL	5.38%	2.14%	1.93%
N801_Tr	14.59%	14.92%	14.59%
N1074_Tr	12.76%	7.16%	6.61%
N1098_Tr	18.39%	15.74%	15.38%
N1158_GC	23.14%	20.18%	19.67%
N1173_aL	19.85%	18.22%	17.79%
N1194_Tr	20.55%	18.99%	18.53%
Average of all sites	16.48%	14.23%	13.77%

Supplementary Table 11: The percentage of galactose as estimated from the LC-MS data.

The apparent relative abundance of each glycan per site was multiplied by the number of HexNac, Man, Gal, Fuc and Neu5Ac residues per glycan. After averaging all sites, the relative % of Gal was calculated from the sum of all the monosaccharides. Please note that this is not a quantitative measure of galactose content.

	***N*-glycans containing galactose**			
	**PRO1-468**	**PRO1-469**	**PRO1-470**	**PRO1-471**
N17_Tr	18.50%	14.31%	16.52%	15.97%
N61_al1	6.43%	8.85%	5.98%	9.85%
N61_al2	7.74%	7.13%	5.69%	10.00%
N61_CH	6.57%	7.93%	5.62%	9.63%
N74_al	19.43%	17.17%	19.71%	17.82%
N122_Tr	14.18%	9.98%	10.96%	13.40%
N149_Gc	18.74%	15.09%	17.13%	17.42%
N165_Tr	19.01%	17.36%	20.10%	18.34%
N165_Gc	20.29%	16.32%	18.67%	18.00%
N234_Tr	0.49%	0.23%	0.27%	0.43%
N282_Tr	17.51%	12.45%	14.90%	16.12%
N331_Gc	16.07%	11.67%	12.62%	15.04%
N343_al	16.96%	11.09%	14.77%	14.12%
N603_al	15.83%	11.16%	12.34%	16.23%
N616_al2	16.16%	11.84%	13.12%	15.05%
N616_al1	16.18%	10.11%	12.45%	13.07%
N657_al1	18.09%	14.10%	16.24%	16.11%
N657_al3	17.12%	14.04%	14.04%	13.38%
N709_al	16.59%	11.80%	13.05%	14.00%
N717_al	4.47%	2.67%	3.28%	4.20%
N801_Tr	9.48%	7.06%	6.83%	9.92%
N1074_Tr	14.06%	7.85%	7.85%	10.84%
N1158_Gc	17.15%	15.39%	14.69%	17.92%
N1173_Gc	20.81%	18.37%	18.12%	19.01%
N1173_Al	20.74%	16.62%	16.01%	19.48%
N1194_Tr	20.44%	18.27%	18.26%	19.57%
Average all sites	15.0%	11.9%	12.7%	14.0%

Supplementary Figure 3. SDS-PAGE Coomassie – Novex gel 4-12%, MES, reducing conditions Final products after buffer exchanged with DPBS pH 7.8. (3 µg per well, Reducing condition: MES buffer, 35 min. at 200 V)

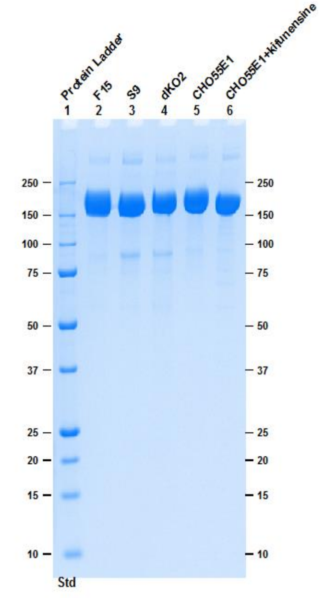

Supplementary Table 12. Pool batches characteristics:

	PRO1-394	PRO1-392	PRO1-412
Tags	C-flag his	C-flag his	C-flag his
Fusion	H-resistin	H-resistin	H-resistin
Storage T°	-80C	-80C	-80C
Transfection method	Stable pool	Stable pool	Stable pool
VCD (E+06 cells/mL)	7.41	9.86	7.09
Viability (%)	95.2	96.5	94.3
Production time (DPI)	11	7	10
Purification	IMAC	IMAC	IMAC
Filtration	0.2um	0.2um	0.2um
Last action	Filtration	Filtration	Centrifugation
Lot comments	Purified from PRO1-7 (C0356, Run 3, same as PRO1-325), buffer exchange using G25, concentration using Amicon Ultra-15 NMWL 50 kDa	Purified from PRO1-389 (C0360, Run 3), buffer exchange using G25, concentration using Amicon Ultra-15 NMWL 50 kDa	Purified from PRO1-413 (C0356, Run 5), concentration and buffer exchange by UF/DF using TFF using Pellicon 3 Biomax 50kDa

Supplementary Methods 1.

For gene disruption by CRISPR/Cas9, separate plasmids encoding *Streptococcus pyogenes* Cas9 (SpCas9) under control of a human elongation factor-1 alpha (EF1α) promoter and single guide RNA (sgRNA) under control of a human U6 promoter were used. The chosen sgRNA target sequences are 5'-AATGAGCATAATCCAACGCCAGG -3' and 5'-ATAAAACAATAAGGTCCCCCAGG-3' for *Fut8* ,as well as 5'-GAAACCTTTTCGTACCTATGGGG-3' and 5'-GCAGTCACGAAAGATCAAGCAGG-3' for *St3gal4.* On the day of transfection, the transfected DNA was a mix of plasmids encoding Cas9_GFP and sgRNAs targeting *Fut8* and/or *ST3Gal*4 at a ratio of 1:1 (w:w) , and the DNA:PEI-Max (polyethylenimine-Max; Polysciences) mixture was prepared at a ratio of 1:8 (w:w). Equal volume of diluted PEI-Max was added to the diluted DNA and incubated for 5 min at room temperature. Finally, polyplexes were added to the cells and the plate was incubated at 37°C and 5% CO2 under constant agitation (120 rpm) for 24 h before fluorescence-activated cell sorting (FACS) for clonal expansion.

Supplementary Methods 2.

For protein expression, cells were maintained in a chemically-defined proprietary media formulation supplemented with 4 mM L-glutamine and incubated in shake flasks (Corning, NY, USA) under agitation (120 rpm) at 37C, 5% CO2. Two days prior to transfection, cells were

seeded in the same media to achieve a cell density of ~8 × 106/mL on the day of transfection. Right before transfection, cells were diluted with 25 % fresh media and dimethylacetamide was added to 0.083 % (v/v). PEI-Max (Polysciences) was used to transfect cells at a DNA:PEI (polyethylenimine) ratio of 1:7 (w:w) and plasmid DNA final concentration was 1.4 µg/mL in cell culture media. The protein-coding sequence was the same as what was used to make the stable pool (see Supplementary Method 3) but cloned into pTT5 instead of pTT241. At 5h post-transfection, 10µM kifunensine was added to the selected cells and at 24h post-transfection, cultures were supplemented with Anti-Clumping Supplement (1:500 dilution) (Irvine Scientific) as well as Feed 4 (2.5 % v/v) (Irvine Scientific) before moving to a 32°C incubator. At 5 days post-transfection, cultures were fed with additional 5% of Feed 4 and additional glucose was added every 2-3 days to maintain a minimal concentration of 17 mM. Finally, cell supernatants were collected at 7 days post-transfection.

Supplementary Methods 3.

Spike trimer was expressed as follows: the SARS-CoV-2 reference strain spike sequence (aa 1-1208 from GenBank accession number MN908947 with the S1/S2 furin site (residues 682–685) mutated [RRAR->GGAS] and K986P / V987P stabilizing mutations was codon-optimized (Cricetulus griseus codon bias) and synthesized by GenScript. To stabilize the spike protein in a trimeric form, the cDNA was cloned in-frame with the human resistin cDNA (aa 23-108) containing a C-terminal FLAG-(His)6 tag (Cricetulus griseus codon bias, GenScript) into the pTT241 expression plasmid and transfected in CHO2353 cells[1] followed by methionine sulfoximine selection for 14 days to generate a stable CHO pool. Cell culture supernatant was harvested 10 days post-induction with cumate and secreted spike trimer present in the clarified medium was purified by immobilized metal-affinity chromatography (Ni-Excel resin; Cytiva). Purified trimeric spike was buffer exchanged in PBS and store as aliquots at -80°C.[1]

Supplementary Methods 4.

Each analyzed sample was unique, resulting in a sample size of 1 per protein. Due to variations in glycan composition on the protein surface for each batch, all samples were considered distinct from one another. No data were excluded from the analysis.

For the HPAEC-PAD analysis, triplicate hydrolyses and injections were performed. The observed standard deviation was compared to the supplier's data and found to be well below the expected threshold. HILIC-Fld and LC-MS analyses were not replicated. In HILIC-Fld, the trace resembled a non-quantifiable fingerprint, and LC-MS and glycopeptide identification were not considered for quantitative studies.

Randomization was not conducted for the study, as it was not relevant due to the uniqueness of each sample (assignment to groups would lack meaningful distinctions), the limited sample size (balancing potential bias would not be significantly beneficial), and analytical reproducibility (the same analysis was replicated multiple times).

The analysts conducting the HPAEC-PAD, HILIC, and LC-MS analyses were blinded. The samples were assigned PRO numbers, ensuring that the analysts were unaware of their identities during the analysis. After data analysis, the samples were compared and identified.

Supplementary Methods 5.

Briefly, MS/MS spectra from each nanoLC-MS/MS DDA raw file were centroided and extracted into mzXML file using ProteoWizard msConvert v3.0 [2]. For identification, each MS/MS spectrum was searched using *m/z* tolerance of 20 ppm against a theoretical spectral library consisting of 2+, 3+ and 4+ ions of peptide range of peptides modified on the asparagine (N) of the N-glycosylation sequon (NXS or NXT). The library search also included a fixed modification of carbamidomethyl on cysteine residues (+57.02 Da) and variable modifications of deamidation on asparagine residues (+0.9840 Da), cyclization to pyro-glutamic acid on N-terminal glutamine residues (-17.027), and cyclization to pyro-glutamic acid on N-terminal glutamic acid residues (-18.011). For quantification, MatchRx [3](a module in GlycoPIQ) was used to extract the *m/z* of all hypothetical glycopeptides in the spectral library (*m/z* tolerance = 10 ppm) in a window +/- 5 min of the glycopeptide expected retention time and calculate the peak area. The sum of the peak areas of 2+, 3+ and 4+ ions was reported as the raw intensity of the glycopeptide. Glycopeptides for which the raw intensity in the non-PNGaseF-treated samples was less than $2^{0.5}$ times the raw intensity in the PNGaseF-treated sample were removed as likely false positives. The raw intensities were normalized, with the abundance of the glycoform for a given peptide with the greatest intensity set at "100%". The abundance of the unmodified peptide (no glycan) was also presented as relative to the most abundant glycoform and so may have an abundance >100%. The GlycoPIQ results then were manually inspected and unusual results were confirmed or removed as false positives (usually due to misannotation of glycopeptides). Final results tables only include glycoforms that have ≥ 5% relative abundance in at least one of the variants. Glycoforms with a relative abundance <1% are listed with an abundance of 0%.

Supplementary References :

1 Joubert, S. *et al.* A CHO stable pool production platform for rapid clinical development of trimeric SARS-CoV-2 spike subunit vaccine antigens. *Biotechnology and Bioengineering* **120**, 1746-1761 (2023). https://doi.org:https://doi.org/10.1002/bit.28387

2 Adusumilli, R. & Mallick, P. in *Proteomics: Methods and Protocols* (eds Lucio Comai, Jonathan E. Katz, & Parag Mallick) 339-368 (Springer New York, 2017).

3 Haqqani, A. S., Kelly, J. F. & Stanimirovic, D. B. in *Genomics Protocols* (eds Mike Starkey & Ramnath Elaswarapu) 241-256 (Humana Press, 2008).
