# Supplementary material for: Simplifying glycan monitoring of complex antigens such as the SARS-CoV-2 spike to accelerate vaccine development
Source: Commun Chem. 2023 Sep 8;6:189. doi: 10.1038/s42004-023-00988-1 (PMC10491790; doi:10.1038/s42004-023-00988-1)
Supplement: Supplementary file 3 — Description of Additional Supplementary Files [file 42004_2023_988_MOESM3_ESM.pdf]

Description of Additional Supplementary Files

File name: Supplementary Data 1

Description: LC-MS data of the glycovariants

File name: Supplementary Data 2

Description: LC-MS data of the 3 stable pool batches
