# Supplementary material for: Simplifying glycan monitoring of complex antigens such as the SARS-CoV-2 spike to accelerate vaccine development
Source: Commun Chem. 2023 Sep 8;6:189. doi: 10.1038/s42004-023-00988-1 (PMC10491790; doi:10.1038/s42004-023-00988-1)
Supplement: Supplementary file 6 — Reporting Summary [file 42004_2023_988_MOESM6_ESM.pdf]

nature portfolio

Corresponding author(s): Janelle Sauvageau

Last updated by author(s): Jul 28, 2023

Reporting Summary

Nature Portfolio wishes to improve the reproducibility of the work that we publish. This form provides structure for consistency and transparency in reporting. For further information on Nature Portfolio policies, see our Editorial Policies and the Editorial Policy Checklist.

Statistics

For all statistical analyses, confirm that the following items are present in the figure legend, table legend, main text, or Methods section.

n/a	Confirmed	
☐	☒	The exact sample size (*n*) for each experimental group/condition, given as a discrete number and unit of measurement
☐	☒	A statement on whether measurements were taken from distinct samples or whether the same sample was measured repeatedly
☐	☒	The statistical test(s) used AND whether they are one- or two-sided<br>*Only common tests should be described solely by name; describe more complex techniques in the Methods section.*
☒	☐	A description of all covariates tested
☒	☐	A description of any assumptions or corrections, such as tests of normality and adjustment for multiple comparisons
☐	☒	A full description of the statistical parameters including central tendency (e.g. means) or other basic estimates (e.g. regression coefficient) AND variation (e.g. standard deviation) or associated estimates of uncertainty (e.g. confidence intervals)
☐	☒	For null hypothesis testing, the test statistic (e.g. *F*, *t*, *r*) with confidence intervals, effect sizes, degrees of freedom and *P* value noted<br>*Give P values as exact values whenever suitable.*
☒	☐	For Bayesian analysis, information on the choice of priors and Markov chain Monte Carlo settings
☒	☐	For hierarchical and complex designs, identification of the appropriate level for tests and full reporting of outcomes
☒	☐	Estimates of effect sizes (e.g. Cohen's *d*, Pearson's *r*), indicating how they were calculated

Our web collection on statistics for biologists contains articles on many of the points above.

Software and code

Policy information about availability of computer code

Data collection	Chromeleon 7 (Thermofisher Scientific) has been used for collecting the monosaccharide data, Xcalibur, version 4.4.16.14 has been used for collecting the HILIC data and the MS data.
Data analysis	Chromeleon 7 (Thermofisher Scientific) has been used for collecting the monosaccharide data and GraphPad Prism 9.0.2 has been used for statistical analysis of the data. Empower 3 (Waters) has been used for analysing the HILIC data, The MS data was analysed using GlycoPIQ v2.1

For manuscripts utilizing custom algorithms or software that are central to the research but not yet described in published literature, software must be made available to editors and reviewers. We strongly encourage code deposition in a community repository (e.g. GitHub). See the Nature Portfolio guidelines for submitting code & software for further information.

Data

Policy information about availability of data

All manuscripts must include a data availability statement. This statement should provide the following information, where applicable:

- Accession codes, unique identifiers, or web links for publicly available datasets
- A description of any restrictions on data availability
- For clinical datasets or third party data, please ensure that the statement adheres to our policy

The data are included in the paper and/ or its supplementary information files. The authors declare that the data supporting the findings of this study are available within the paper and its supplementary information files.

Human research participants

Policy information about studies involving human research participants and Sex and Gender in Research.

Reporting on sex and gender	No human research participants were involved.
Population characteristics	No human research participants were involved.
Recruitment	No human research participants were involved.
Ethics oversight	No human research participants were involved.

Note that full information on the approval of the study protocol must also be provided in the manuscript.

Field-specific reporting

Please select the one below that is the best fit for your research. If you are not sure, read the appropriate sections before making your selection.

☒ Life sciences ☐ Behavioural & social sciences ☐ Ecological, evolutionary & environmental sciences

For a reference copy of the document with all sections, see nature.com/documents/nr-reporting-summary-flat.pdf

Life sciences study design

All studies must disclose on these points even when the disclosure is negative.

Sample size	Each sample analysed was different from another and thus, per protein the sample size was of 1. As every batch produced different glycans on the surface of the protein, each sample is considered different from the other.
Data exclusions	No data was excluded
Replication	For the HPAEC-PAD analysis, triplicates hydrolyzes were performed and triplicate injections of each hydrolyzes were performed. The standard deviation observed was compared to the supplier standard deviation data and were well below the threshold expected. For HILIC-Fld and LC-MS, no replicates were performed. For HILIC-Fld, the trace ressembled a non-quantifyable fingerprint, the nature of LC-MS and glyco-peptide identification is not considered for quantitative studies.
Randomization	Randomization was not performed for the study as not relevant due to sample uniqueness (each sample is unique, assigning samples to groups would not be meaningful), limited sample size (it does not provide significant benefit in terms of balancing potential bias) and analytical reproducibility (we replicated the same analysis multiple times).
Blinding	The analysts performing the analyses was blinded for HPAEC-PAD, HILIC and LC-MS. Indeed, the samples have been assigned PRO numbers that the analysts did not know the identity of the samples. After analysis of the data the samples were compared and identified.

Reporting for specific materials, systems and methods

We require information from authors about some types of materials, experimental systems and methods used in many studies. Here, indicate whether each material, system or method listed is relevant to your study. If you are not sure if a list item applies to your research, read the appropriate section before selecting a response.

Materials & experimental systems

n/a	Involved in the study	
☒	☐	Antibodies
☐	☒	Eukaryotic cell lines
☒	☐	Palaeontology and archaeology
☒	☐	Animals and other organisms
☒	☐	Clinical data
☒	☐	Dual use research of concern

Methods

n/a	Involved in the study	
☒	☐	ChIP-seq
☒	☐	Flow cytometry
☒	☐	MRI-based neuroimaging

Eukaryotic cell lines

Policy information about cell lines and Sex and Gender in Research

Cell line source(s)	Cricetulus griseus from a female hamster
Authentication	Genome sequencing
Mycoplasma contamination	Negative
Commonly misidentified lines<br>(See ICLAC register)	None
